# Relationship Between Cerebral Oximetry and Outcomes in Post-Cardiac Arrest Patients: A Systematic Review and Meta-Analysis

**DOI:** 10.1097/CCE.0000000000001427

**Published:** 2026-07-02

**Authors:** Atul Phillips, Caralyn Bencsik, Ish Bains, Erica McKenzie, Andy Wong, Philippe Couillard, Julie A. Kromm, Andreas H. Kramer

**Affiliations:** 1 Department of Critical Care Medicine, Christian Medical College, Ludhiana, Punjab, India.; 2 Department of Critical Care Medicine, University of Calgary, Calgary, AB, Canada.; 3 Alberta Health Services, Calgary, AB, Canada.; 4 Department of Clinical Neurosciences, University of Calgary, Calgary, AB, Canada.; 5 Hotchkiss Brain Institute, Calgary, AB, Canada.

**Keywords:** brain, heart arrest, hypoxia, out-of-hospital cardiac arrest, post-cardiac arrest syndrome

## Abstract

**OBJECTIVES:**

Poor neurologic outcomes are common in post-cardiac arrest patients. Reduced cerebral perfusion and inadequate oxygen delivery may persist even following return of spontaneous circulation (ROSC), which may exacerbate hypoxic-ischemic brain injury. Cerebral oximetry is a noninvasive method of assessing brain oxygenation. We performed a systematic review to assess whether post-arrest regional oxygen saturation (rSO_2_) during the initial 48 hours post-ROSC is associated with death or severe disability.

**DATA SOURCES:**

MEDLINE, Embase, Cochrane Central Register of Controlled Trials, World Health Organization Global Health Library.

**STUDY SELECTION:**

We included studies reporting outcomes in adult post-arrest patients who underwent continuous cerebral oximetry immediately following ROSC for at least 6 hours.

**DATA EXTRACTION:**

rSO_2_ over the 48 hours post-arrest was compared between patients with favorable vs. unfavorable outcomes, as dichotomized within individual studies.

**DATA SYNTHESIS:**

Random effects models were used to pool studies. 10,876 records were identified, of which 16, with a total of 864 patients, met inclusion criteria. Twelve studies provided neurologic outcomes, and four reported only survival. rSO_2_ was similar at baseline, but higher at 24 hours (3.3%, 0.9–5.7%, *p* = 0.009) and 48 hours (2.1%, 0.3–3.9%, *p* = 0.02) post-arrest among patients with favorable outcomes. There was significant heterogeneity between studies. rSO_2_ averaged over time was significantly higher between 24 and 48 hours in patients with favorable outcomes (5.2%, 1.6–8.8%; *p* = 0.005). rSO_2_ was lower in studies where cardiac arrest duration was relatively longer (modeled difference –7.1%, –3.1% to –11.1%, *p* = 0.0005). Although very low rSO_2_ had high specificity for unfavorable outcome in two studies, no consistent threshold could be identified for use in neuroprognostication.

**CONCLUSIONS:**

Current evidence suggests that rSO_2_ during the initial 48 hours following ROSC is slightly lower in post-cardiac arrest patients with unfavorable neurologic outcomes (low certainty of evidence). Prospective research is needed to determine whether treatment of reduced rSO_2_ can improve outcomes.

**KEY POINTS**

**Question:** Is reduced regional oxygen saturation (rSO_2_) following resuscitation from cardiac arrest associated with worse neurologic outcomes?

**Findings:** This systematic review identified 16 studies with 864 patients and found that rSO_2_ at 24 and 48 hours and rSO_2_ averaged between 24 and 48 hours were lower among patients with poor outcomes, although differences were small and of unclear clinical significance. rSO_2_ was lower in studies with longer cardiac arrest duration.

**Meaning:** The role of cerebral oximetry in management of post-arrest patients remains unclear. Further research is needed to determine whether correction of low rSO_2_ can improve outcomes.

Out-of-hospital cardiac arrest has an annual incidence of more than 80 cases per 100,000 population (1, 2). About a quarter of patients achieve return of spontaneous circulation (ROSC), and only about 10% survive to hospital discharge (1, 2). In-hospital cardiac arrest has an incidence of 1–10 cases per 1000 hospital admissions and a survival rate of about 20% (3).

Hypoxic-ischemic brain injury (HIBI) is the most common cause of death and long-term disability in patients resuscitated from cardiac arrest (4). The neurovascular unit is vulnerable to oxygen deprivation. Primary injury occurs during periods of “no flow” and “low flow.” Following ROSC, there may be reperfusion injury, with contributing factors including endothelial dysfunction, free radical formation, glutamate release, accumulation of intracellular calcium, nitric oxide depletion (with resultant vasoconstriction), microthrombus formation, blood–brain barrier disruption, and loss of autoregulation. A transient period of cerebral hyperemia is followed by hypoperfusion and ongoing vulnerability to secondary brain injury (5–11). Brain injury may be perpetuated further by impaired diffusion of oxygen and other mechanisms that interfere with oxygen usage (12).

Despite potentially inadequate perfusion and tissue oxygenation, clinical trials assessing measures aimed at augmenting cerebral oxygen delivery, including higher mean arterial pressure, increased Pao_2_, and hypercapnia-induced cerebral vasodilatation have not demonstrated improved outcomes (13–15). These interventions were applied using fixed therapeutic targets irrespective of whether cerebral oxygen delivery was impaired. A more targeted approach would be for physiologic goals to be guided by neuromonitoring (16). Invasive brain tissue oxygen tension or cerebral blood flow monitors are rarely inserted in post-arrest patients. Patients where the arrest is due to cardiac etiologies often require antiplatelet therapy or anticoagulation, which contraindicate placement of intracranial devices. Jugular bulb oxygen saturation is measured at some centers but requires placement of a retrograde catheter into the internal jugular vein, maintained at approximately the level of C1 vertebra (17).

In contrast, cerebral oximetry is a safe, noninvasive, and user-friendly modality that uses near-infrared spectroscopy (NIRS) to assess hemoglobin oxygen saturation in the frontal lobes. Probes are applied to the forehead and emit light with wavelength in the near infrared spectrum (650–1000 nm), to which bone is relatively transparent. Light is absorbed by chromophores, including hemoglobin, according to the Beer-Lambert principle, whereby absorption is greater with a higher concentration of a substance and increasing distance. Reflected light is measured at the scalp by sensors in the probe. The light absorption spectrum differs for oxyhemoglobin and deoxyhemoglobin. By emitting light of multiple wavelengths and measuring the reflection, commercially available oximeters can estimate hemoglobin oxygen saturation. Depth is controlled through the distance between light emitters and detectors. Cerebral oximetry assesses mainly venous blood, such that regional oxygen saturation (rSO_2_) is usually 60–75% (18, 19). Low preoperative rSO_2_ and intraoperative desaturation during cardiac surgery have been associated with postoperative cognitive impairment (20–22). Desaturation during extracorporeal life support is predictive of cerebral ischemia (23). Higher rSO_2_ during cardiopulmonary resuscitation has been associated with a greater chance of ROSC (24).

Cerebral oximetry has also been used in post-cardiac arrest patients. A limitation in this setting is that, unlike operating room patients, baseline rSO_2_ values are unavailable. We performed a systematic review to assess whether lower rSO_2_ during the initial 48 hours post-arrest is associated with death or poor neurologic recovery.

## METHODS

This review was registered with PROSPERO (CRD42024602416) and was conducted in accordance with the Preferred Reporting Items for Systematic Review and Meta-Analysis guidelines (25).

The population of interest was adult patients (older than 18 yr) immediately following resuscitation from either out-of-hospital or in-hospital cardiac arrest. To be included, manuscripts could be either randomized controlled trials or cohort studies, and needed to initiate cerebral oximetry immediately following ROSC and admission to the ICU, followed by continuous monitoring for at least 6 hours. We did not include studies where cerebral oximetry was assessed only during cardiopulmonary resuscitation or when it was initiated more than six hours after ICU admission. We also excluded case reports or studies with fewer than 10 patients.

Our goal was to assess the association between rSO_2_ levels during the initial 48 h post-arrest and neurologic outcomes. We anticipated that publications would describe outcomes inconsistently, and therefore planned to pool data based on the method of dichotomization between favorable and unfavorable outcomes, as reported in individual studies. The most common method of reporting post-arrest outcomes is the Cerebral Performance Category (CPC) scale; we considered 1 (full recovery or mild disability) or 2 (moderate disability but independent in activities of daily living) to be favorable and 3 (severe disability; dependent in activities of daily living), 4 (persistent vegetative state), or 5 (dead) to be unfavorable. If an alternative scoring system was used, we planned to dichotomize outcomes in an analogous manner (e.g., Glasgow Outcome Scale (extended) 5–8 vs. 1–4, or modified Rankin scale 0–3 vs. 4–6). If an article reported only mortality, we considered survival to be a favorable outcome, but also conducted separate sensitivity analyses specifically involving studies reporting neurologic outcomes, acknowledging that some patients may survive with severe disability (3). The last available outcome provided in each article up to 12 months post-arrest was used.

For our primary analysis, we compared differences in mean rSO_2_ values between patients with favorable and unfavorable outcomes, both at individual timepoints and as a continuous variable averaged over time. In studies where rSO_2_ was measured bilaterally, the average between the two sides was used. We also assessed whether any rSO_2_ threshold consistently predicted neurologic prognosis with high diagnostic accuracy. Additional exploratory aims included evaluating the association between arrest details (duration or initial rhythm) or post-arrest treatments (therapeutic hypothermia, defined as temperature ≤ 34°C for 24 hr) and rSO_2_. We further assessed effect modification of these variables, as well as study design (randomized vs. observational), on the association between rSO_2_ and outcomes. Collection of all data was planned a priori, although the exploratory analyses based on arrest details and study design were not prespecified in our PROSPERO protocol.

A medical librarian was consulted to assist with the review. We searched the English language literature, using the following databases from their inception until March 17, 2026: MEDLINE, Embase, Cochrane Central Register of Controlled Trials, Cochrane Database of systematic reviews, and the World Health Organization Global Health Library. The search was organized using three domains: “cerebral oximetry,” “cardiac arrest,” and “outcomes.” For each domain, relevant Medical Subject Heading terms were identified and incorporated into the search together with related keywords. The domains were combined using the Boolean operator “AND.” Study selection was performed with Covidence software (Melbourne, Australia). Title and abstract screening, initial selection of papers for full review, final study selection, data abstraction, and risk of bias assessments were all conducted by two independent reviewers, with disagreements resolved by a third reviewer. Risk of bias was determined using the Quality Assessment of Diagnostic Accuracy Studies-2 tool. A risk of bias due to missing evidence table was used to summarize missing data.

Patient characteristics across studies were assessed with descriptive statistics. Generalized estimating equation models, with terms for outcome (favorable vs. unfavorable), time (hours), and an interaction term were used to model rSO_2_ over time, as reported in individual studies, using an autoregressive correlation structure. Data were combined and meta-analyzed at individual time points (baseline, 24 hr, and 48 hr) and as averages over time (initial 24 hr and 24–48 hr), using random effects models to quantify differences in means (including 95% CIs) and Forrest plots to summarize results. Study weights were assigned using the inverse of variance. For studies reporting median values, we divided the interquartile range by 1.35 to estimate the sd, as recommended in the Cochrane Handbook (26). Because manuscripts did not report individual patient data, we divided studies in half based on average cardiac arrest duration and compared results above and below the median. A similar approach was used to assess outcomes based on the proportion of patients with shockable rhythms or treated with therapeutic hypothermia. Effect modification was assessed using random effects models. Q values and *I*^2^ were calculated to assess heterogeneity across studies. Funnel plots and Egger regression intercept assessed publication bias. A *p* value of less than 0.05 was considered statistically significant. Analyses were conducted using SAS 9.4 (Cary, NC) and Comprehensive Meta-analysis 4 (Englewood, NJ). Graphpad Prism 10.1 (Boston, MA) was used to create figures. The Grading of Recommendations, Assessment, Development, and Evaluation framework was used to rate the strength of findings and describe our key conclusions (27).

## RESULTS

### Study Selection

**Figure 1.**
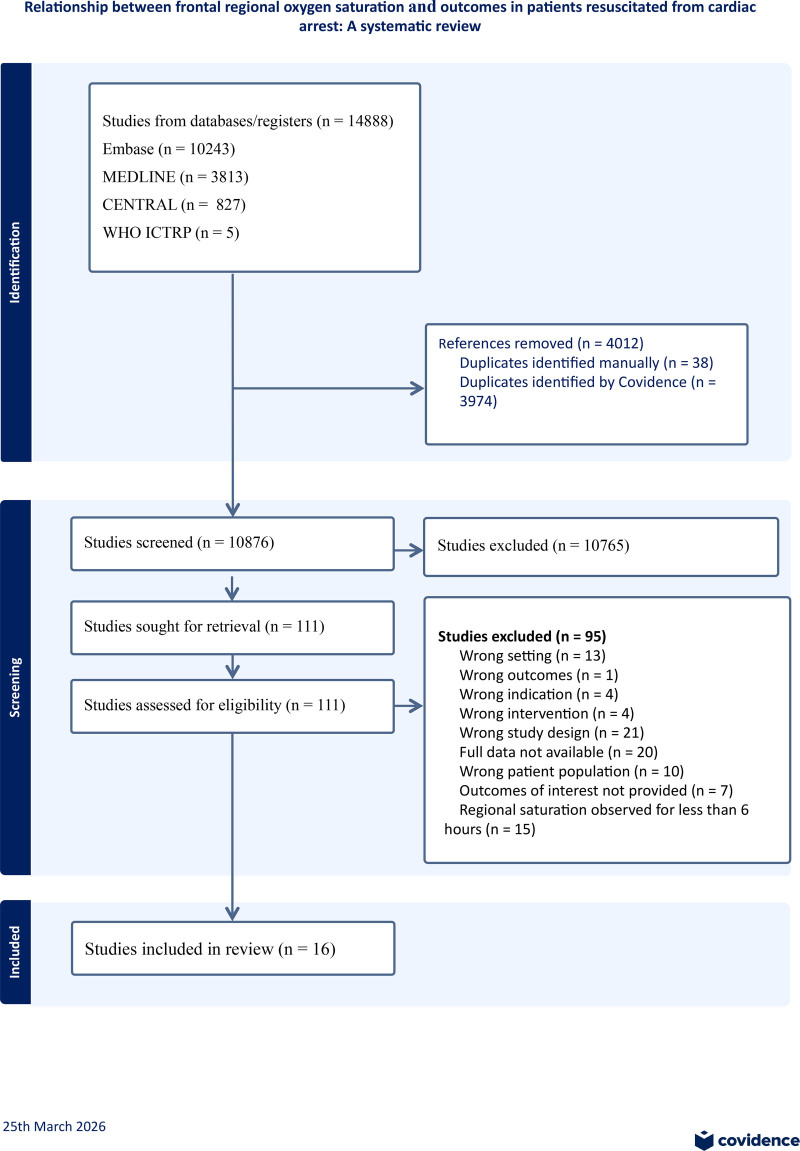
Flowsheet for identification of studies assessing association between cerebral oximetry and outcomes in post-cardiac arrest patients. CENTRAL = Cochrane Central Register of Controlled Trials, WHO ICTRP = World Health Organization International Clinical Trials Registry Platform.

A flowsheet for article selection is shown in **Figure 1**. Medline search results are included in Supplementary Files (**Appendix**, https://links.lww.com/CCX/B645). After removal of duplicates, the search revealed 10,876 records. The final analysis included 16 studies with a total of 864 patients.

### Risk of Bias

There was potential risk of bias based on inconsistent reporting of rSO_2_ at different timepoints post-arrest, although it was reported at baseline and 24 hours post-arrest in 13 of 16 studies and at 48 hours in 12 of 16 studies. Reporting of average rSO_2_ over time (0–24 hr and 24–48 hr) occurred in fewer than one-half of studies (**Table e1**, https://links.lww.com/CCX/B645). Egger regression analysis did not reveal significant evidence of publication bias for baseline, 24-, or 48-hour rSO_2_, although the Funnel plot at 48 hours did suggest a greater difference in rSO_2_ in studies with higher se **(Figs. e1–e3**, https://links.lww.com/CCX/B645). Risk of bias in inpatient selection was assessed as being low, with studies generally enrolling consecutive patients meeting inclusion criteria (**Table e2**, https://links.lww.com/CCX/B645). There was variability of inclusion and exclusion criteria, with some studies limited to either in- or out-of-hospital cardiac arrest, patients with shockable rhythms, or those with a cardiac etiology for the arrest. Overall, however, the review included a diverse patient population, broadly representative of cardiac arrest patients who are admitted to multisystem ICUs. Measurement of rSO_2_ occurred before assessment of prognosis and was unlikely to have been interpreted differently based on anticipated outcome. Studies did not provide information about how decisions regarding withdrawal of life-sustaining measures were made, but we considered it unlikely that this was influenced by rSO_2_, considering that cerebral oximetry is not recommended for this purpose in major guidelines (28–31). Risk of bias related to study flow and timing was deemed to be low, although **4** studies reported only mortality and not neurologic outcome.

### Study and Patient Characteristics

Study and patient characteristics are shown in **Table 1** and **Table e3** (https://links.lww.com/CCX/B645). There were 14 prospective cohort studies and 2 randomized controlled trials, performed in 12 different countries (32–47). Cerebral oximetry products included Invos (9; Medtronic, Minneapolis, MN), Foresight (4; Edwards, Irvine, CA), and Equinox or Sensmart (3; Nonin, Plymouth, MN) devices, and the duration of monitoring ranged from 24 hours to 5 days. Three studies published data from the same growing dataset; for the meta-analysis, we used only the most recent publication (32, 35, 39). Some relevant studies were excluded because they involved only pediatric patients or initiated cerebral oximetry many hours after ROSC (48–51).

**TABLE 1.** Characteristics of Studies Assessing the Relationship Between Cerebral Regional Oxygen Saturation and Outcome

Study	Design (*n*)	Age (sd or Interquartile Range)	Out-of-Hospital Cardiac Arrest (%)	Ventricular Fibrillation/Ventricular Tachycardia (%)	Arrest Minutes (sd or Interquartile Range)	Target Temp (°C)	Outcome and Timing
Meex et al (32)	Cohort (28)	61 (14)	100	75	22 (15)	33	Hospital mortality
Ahn et al (33)	Cohort (21)	65 (58–74)	38	NR	NR	NR	Hospital mortality
Storm et al (34)	Cohort (60)	68 (56–74)	63	50	16 (8–29)	33	CPC 6 mo
Ameloot et al (35)^a^	Cohort (82)	63 (13)	NR	66	21 (16)	33	CPC 6 mo
Pham et al (36)	Cohort (23)	64 (49–78)	74	30	34 (15–62)	36	CPC 3 mo
Ibrahim et al (37)	Cohort (19)	66 (12)	0	10	19 (NR)	NR	Hospital mortality
Bougle et al (38)	Cohort (43)	60 (13)	100	81	23 (NR)	33	CPC hospital discharge
Genbrugge et al (39)^a^	Cohort (107)	63 (13)	100	62	30 (19)	33	Hospital mortality
Saritas et al (40)	Cohort (25)	50 (17)	NR	NR	18 (18)	35	CPC hospital discharge
Jakkula et al (41)	RCT^b^ (118)	60 (13)	100	100	22 (19–26)	33 or 36	CPC 6 mo
Tran et al (42)	Cohort (87)	64 (NR)	0	15	11 (NR)	NR	CPC hospital discharge
Kwon et al (43)	RCT^b^ (57)	69 (13)	100	21	29 (18)	33 or 36	CPC 6 mo
Sakurai et al (44)	Cohort (49)	65 (15)	100	37	31.7 (19)	34	CPC hospital discharge
Schnaubelt et al (45)	Cohort (27)	59 (53–73)	100	52	21 (14–31)	32–34	CPC hospital discharge
Ryu et al (46)	Cohort (78)	58 (17)	100	33	22 (13–37)	33 or 36	CPC 6 mo
Yazar et al (47)	Cohort (40)	58 (46–70)	78	43	27 (15–38)	34	CPC 1 mo

CPC = Cerebral Performance Category, NR = not reported.

a These studies used the same dataset, such that only most recent data used for meta-analysis.

b Jakkula et al (41) compared different physiologic targets. Kwon et al (43) compared management at 33 vs. 36°C.

Mean patient age across studies was 62 years and 628 (73%) were male. When location of cardiac arrest was reported, 79% were out-of-hospital and 21% in-hospital. When initial rhythm was reported, it was shockable (ventricular fibrillation or ventricular tachycardia) in 53% of cases. Average cardiac arrest duration ranged from 11 to 34 minutes. Goal temperature during the initial 24 hours varied between 32 and 34°C (8), either 33°C or 36°C (3), 35°C (1), 36°C (1), and not reported (3).

Four studies compared rSO_2_ between survivors and non-survivors only, without reporting data about functional outcomes in survivors (32, 33, 37, 39). In these studies, hospital mortality ranged from 36% to 72%. Twelve studies provided CPC scores, with outcomes adjudicated at hospital discharge in seven studies and after 3–6 months in five studies (34–36, 38, 40–47). No other outcome scales were used. The proportion of patients with favorable neurologic recovery ranged from 23% to 66%. Fourteen studies reported rSO_2_ values at various time points post-arrest (Table e1, https://links.lww.com/CCX/B645) (32, 34, 37–47). Seven studies reported average rSO_2_ values over time (33, 34, 36–39, 41). Five studies attempted to identify a threshold to predict outcome (34, 38, 39, 43, 45). Five manuscripts explicitly mentioned excluding patients with acute or chronic brain injury (36, 38–40, 43).

### Association Between rSO_2_ and Outcomes

**Figure 2.**
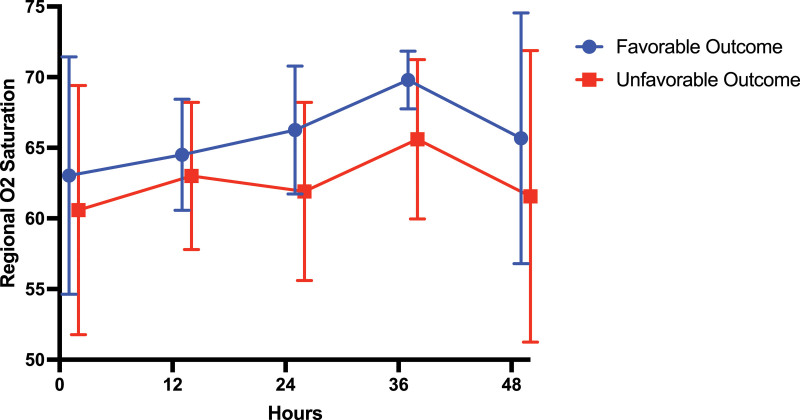
Regional oxygen saturation over time in studies involving post-cardiac arrest patients.

rSO_2_ during the initial 48 hours post-cardiac arrest was higher among patients with favorable outcomes, although differences were numerically small (**Fig. 2**). The sd at 12 to 36 h was larger for patients with unfavorable outcomes, indicating a greater tendency to have low rSO_2_, but also that many patients with poor outcomes had normal or higher values. rSO_2_ is generally lowest during the initial hours post-arrest and then increases over the subsequent 24–48 hours.

**Figure 3.**
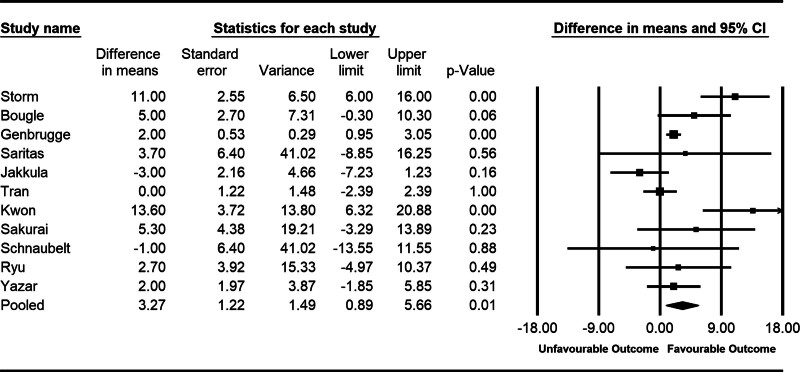
*Forrest plot* showing difference in regional oxygen saturation at 24 hours after arrest between post-cardiac arrest patients with unfavorable vs. favorable outcomes.

**Figure 4.**
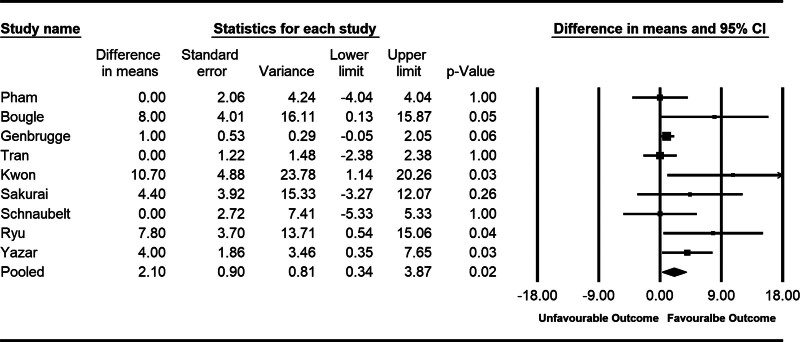
*Forrest plot* showing difference in regional oxygen saturation at 48 hours post-cardiac arrest in patients with unfavorable vs. favorable outcomes.

Meta-analysis found baseline rSO_2_ across studies to be nonsignificantly higher in patients with favorable outcomes (1.6%, –1.4% to 4.7%, *p* = 0.29; **Fig. e4**, https://links.lww.com/CCX/B645). rSO_2_ at 24 h was higher in patients with favorable outcome (3.3%, 0.9–5.7%, *p* = 0.009; **Fig. 3**), but there was marked heterogeneity between studies (Q value 32.4, *p* < 0.001; *I*^2^ 69%). rSO_2_ was also higher at 48 hours (2.1%, 0.3–3.9%, *p* = 0.02; **Fig. 4**), but with less heterogeneity (Q value 14.4, *p* = 0.07; *I*^2^ 44%). When we excluded one study reporting only mortality and not functional outcomes, the significant difference in rSO_2_ persisted at both 24 and 48 hours (39).

rSO_2_ averaged over the initial 24 hours post-arrest was nonsignificantly higher in patients with favorable outcomes (**Fig. e5**, https://links.lww.com/CCX/B645; 2.4%, –0.4% to 5.1%, *p = 0*.09) (33, 34, 36, 38, 39). rSO_2_ averaged from 24 to 48 hours post-arrest was significantly higher in patients with unfavorable outcomes (**Fig. e6**, https://links.lww.com/CCX/B645; 5.2%, 1.6–8.8%, *p* = 0.005). There was no significant heterogeneity for this analysis (Q value 4.5, *p* = 0.22; *I*^2^ 33%), but it was based on only four studies that reported rSO_2_ averaged over this timeframe (33, 34, 36, 38).

Given the inconsistent reporting of rSO_2_ at different timepoints in the 48 hours post-arrest and substantial heterogeneity of results, we graded the evidence that low rSO2 is associated with poor neurologic outcome in post-arrest patients to be of low certainty.

### Assessment of Effect Modification

Results of effect modification assessments are shown in **Table e4** (https://links.lww.com/CCX/B645). The difference in rSO_2_ between patients with favorable vs. unfavorable outcomes was statistically significant at 24 hours only in studies with relatively longer (4.5%, 0.8–8.1%, *p* = 0.02), but not shorter, cardiac arrest duration (22–32 min vs. 11–21 min), and in studies where patients were routinely treated with hypothermia in the first 24 hours (4.3%, 0.7–7.9%, *p* = 0.02), but not at higher temperatures. However, random effects models did not find any significant evidence of effect modification between subgroups.

### Subgroup Analysis

**Figure 5.**
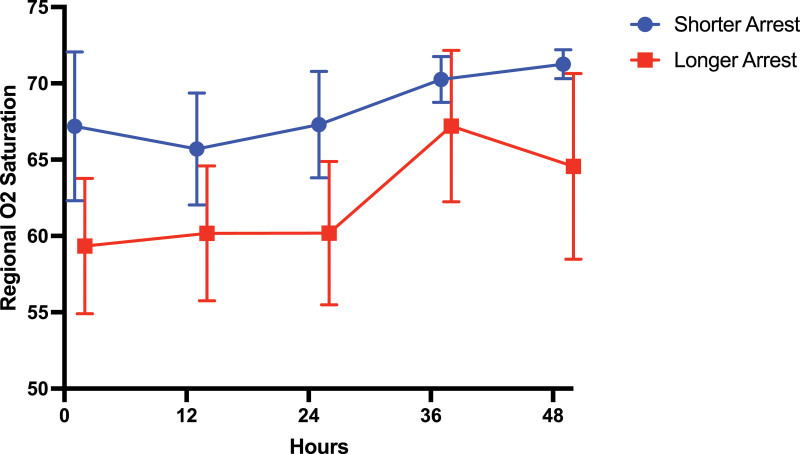
Comparison of regional oxygen saturation over time in studies with shorter vs. longer mean cardiac arrest duration.

Studies with longer cardiac arrest duration had lower rSO_2_ values when modeled over 48 hours post-arrest (–7.1%, –3.1% to –11.1%, *p* = 0.0005; **Fig. 5**). In studies where patients were routinely treated with therapeutic hypothermia (≤ 34°C), rSO_2_ was lower at 12 hours post-arrest (61.8% vs. 66.8%, *p* = 0.04), but did not differ significantly when modeled over 48 hours (–3.7%, –2.3% to 9.8%, *p* = 0.22; **Fig. e7**, https://links.lww.com/CCX/B645). There was no significant difference in rSO_2_ between studies with a higher vs. lower proportion of patients with shockable rhythms (average difference 0%, –6.8% to 6.9%, *p* = 0.99; **Fig. e8**, https://links.lww.com/CCX/B645).

### rSO_2_ Thresholds in Outcome Prediction

When reported, area under the curve for rSO_2_ in the prediction of death or poor neurologic recovery outcome ranged from 0.58 to 0.80 (33, 38, 42). Storm and Genbrugge et al (34) found cumulative time below a rSO_2_ of 50% and 55%, respectively, to be predictive of poor outcome (39). Bougle et al (38) reported specificity of 100% (with sensitivity 45%) in prediction of poor outcome for any rSO_2_ less than 30%. Kwon et al (43) reported specificity of 100% (with sensitivity 45%) when the 24-hour rSO_2_ was less than 48.8%.

## DISCUSSION

We found that rSO_2_ was slightly higher in post-cardiac arrest patients with favorable neurologic outcomes at 24- and 48 hours following ROSC, although differences compared with patients with unfavorable outcomes were small and of unclear clinical importance. The sd of rSO_2_ was wider among patients with poor outcomes. Because of significant heterogeneity between studies, we graded the evidence for an association between rSO_2_ and outcomes as being of low certainty. There was no effect modification based on cardiac arrest duration, initial rhythm, or use of therapeutic hypothermia. rSO2 at 24 and 48 hours was consistently lower in studies with longer cardiac arrest duration. We could not identify a specific rSO_2_ level that was consistently predictive of poor neurologic outcomes.

Outcomes are poor for a large proportion of post-cardiac arrest patients. There are currently no treatments known to improve neurologic recovery. Although promising in early clinical trials, therapeutic hypothermia is currently not endorsed by International Liaison Committee on Resuscitation (ILCOR) guidelines because the largest and highest quality studies did not show efficacy (52–55). Studies seeking to augment cerebral oxygen delivery with higher mean arterial pressure, Po_2_, or induced hypercapnia have similarly not shown any improvements in outcomes (13–15). There has, however, been a paucity of trials individualizing physiologic targets based on neuromonitoring information. Although invasive neuromonitoring has been used in HIBI, it is impractical for widespread use, and many patients have contraindications (2, 56). In contrast, cerebral oximetry is widely accessible, noninvasive, safe, and easy to use (18, 19).

Our observations support the hypothesis that maintaining rSO_2_ levels above a certain level could lead to a greater chance of favorable recovery. However, it also remains possible that low rSO_2_ is a marker of more severe HIBI rather than a modifiable indicator of secondary brain injury. Lower rSO_2_ in post-arrest patients may be attributable to the pathophysiological consequences of ischemia-reperfusion injury, such as vasoconstriction, microthrombus formation, and disturbed autoregulation, which may persist for longer in patients with poor outcomes (5–11). The finding that rSO_2_ post-arrest was consistently lower in studies where average cardiac arrest duration was relatively longer further supports the possibility that lower rSO_2_ may be a characteristic of more severe HIBI. Lower rSO_2_ may, in some cases, also be due to physiologic derangements, such as lower cardiac output and blood pressure, rather than necessarily more severe HIBI.

Studies have shown that diffusion and cellular utilization of oxygen are impaired with HIBI, such that more severe injury could also be expected to elevate rSO_2_ levels (12, 56, 57). It is therefore possible that both very low and high rSO_2_ values may be associated with poor outcomes, which may explain the larger sd in rSO_2_ among patients with unfavorable outcomes and may also have attenuated the overall association between average rSO_2_ and outcomes. Lower rSO_2_ can be treated by augmenting cerebral oxygen delivery. The degree to which high rSO_2_ can or should be treated is less clear. A recent study using jugular bulb venous oximetry found that venous saturation decreased (arteriovenous difference increased) with administration of hypertonic saline, suggesting that treatment of cerebral edema may improve diffusion of oxygen and increase utilization (58).

There was heterogeneity in the association between rSO_2_ and outcomes, with some studies finding lower rSO_2_ to be strongly predictive of poor outcomes (34, 43), and others reporting no relationship (36, 41, 42). Reasons for this heterogeneity are not clear but results may have been affected by differing patient characteristics (duration of cardiac arrest, type of rhythm, degree of physiologic instability), and management (provision of bystander cardiopulmonary resuscitation, use of therapeutic hypothermia). Although rSO_2_ was consistently lower among patients with relatively longer cardiac arrest duration and was lower during the initial 12–24 hours post-arrest among patients treated with therapeutic hypothermia, we found no definitive effect modification for the relationship between rSO_2_ and outcomes. rSO_2_ may also be influenced by factors such as skin pigmentation, age, skull thickness, and hemoglobin concentration, which were not reported in most studies (59–61).

Diagnostic tests with high specificity for unfavorable outcomes are helpful for informing goals of care conversations with families of post-arrest patients (28–31). rSO_2_ below 30–50% appeared to be predictive of poor outcome with 100% specificity in two studies (38–43). However, there was no consistent threshold identified for rSO_2_ reduction currently to be useful in neuroprognostication. Whether very low rSO_2_ values can, in the future, be used to assist with multimodal neuroprognostication requires further study.

This systematic review has important limitations. Some studies reported rSO_2_ values at individual time points, whereas others reported values averaged over time. The specific timepoints where rSO_2_ was reported varied between studies. Some studies reported only mortality and not functional recovery. Given that clinicians were generally not blinded to cerebral oximetry, it is unclear how awareness of rSO_2_ may have modified patient management. Our study did not address the utility of cerebral oximetry for predicting ROSC during cardiopulmonary resuscitation or assessing CBF autoregulation and optimizing blood pressure targets, both of which have been assessed in other systematic reviews (62, 63).

## CONCLUSIONS

In conclusion, current evidence suggests that rSO_2_ during the initial 48 hours following ROSC is slightly lower in post-cardiac arrest patients who have unfavorable neurologic outcomes (low certainty of evidence). Further research is required to determine whether treatment of rSO_2_ reductions can improve outcomes.

## Supplementary Material
