# Supplementary material for: Relationship Between Cerebral Oximetry and Outcomes in Post-Cardiac Arrest Patients: A Systematic Review and Meta-Analysis
Source: Crit Care Explor. 2026 Jul 2;8(7):e1427. doi: 10.1097/CCE.0000000000001427 (PMC13331409; doi:10.1097/CCE.0000000000001427)
Supplement: Supplementary file 1 [file cc9-8-e1427-s001.pdf]

Supplementary Files (Tables e1-e4, Figures e1-e8, Appendix)

Table e1. Risk of Bias due to Missing Evidence Table for reported outcomes.

Study (Ref)	Baseline RSO2	RSO2 at 24 hours	RSO2 at 48 hours	Average RSO2 over 24 hours	Average RSO2 24-48 hours
Meex (32)[1] Ameloot (35)[1] Genbrugge (39)[1]	Yes	Yes	Yes	Yes	No
Ahn (33)	No	No	No	Yes	Yes
Storm (34)	Yes	Yes	No	Yes	Yes
Pham (36)	No	No	Yes	Yes	Yes
Ibrahim (37)	Yes	No	No	No	No
Bougle (38)	No	Yes	Yes	Yes	Yes
Saritas (40)	Yes	Yes	No	No	No
Jakkula (41)	Yes	Yes	No	No	No
Tran (42)	Yes	Yes	Yes	No	No
Kwon (43)	Yes	Yes	Yes	No	No
Sakurai (44)	Yes	Yes	Yes	No	No
Schnaubelt (45)	Yes	Yes	Yes	No	No
Yazar (46)	Yes	Yes	Yes	No	No

[1]These studies utilized the same dataset, such that only the most recent data was used for meta-analysis

Figure e1. Funnel plot showing relationship between standard error and difference in means for studies reporting RSO_2 at start of monitoring.

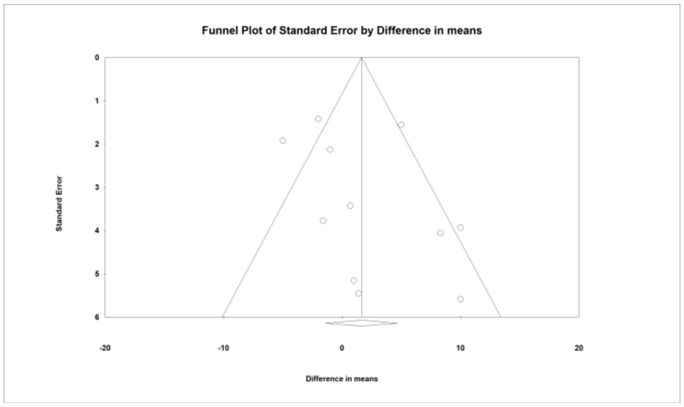

Egger's regression intercept 1.39 (-1.30 to 4.07), p=0.27

Figure e2. Funnel plot showing relationship between standard error and difference in means for studies reporting RSO_2 at 24 hours post-cardiac arrest.

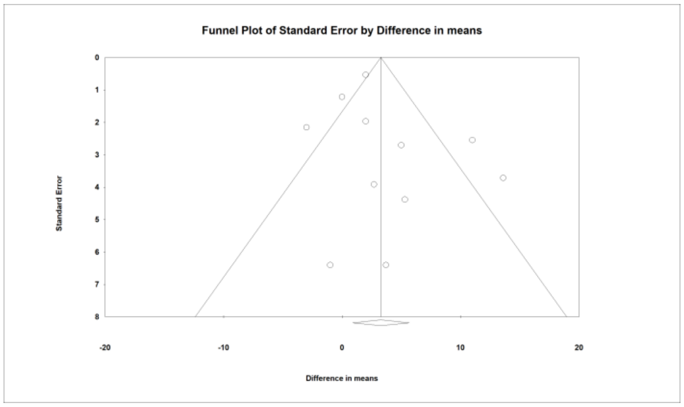

Egger's regression intercept 0.79 (-0.99 to 2.58), p=0.34

Figure e3. Funnel plot showing relationship between standard error and difference in means for studies reporting RSO_2 at 48 hours post-cardiac arrest.

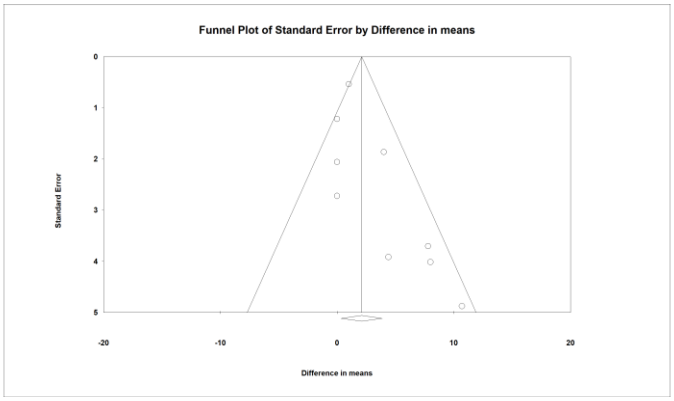

Egger's regression intercept 1.27 (-0.01 to 2.54), p=0.05

Table e2. Risk of bias assessment in studies assessing association between RSO_2 and outcomes in post-cardiac arrest patients.

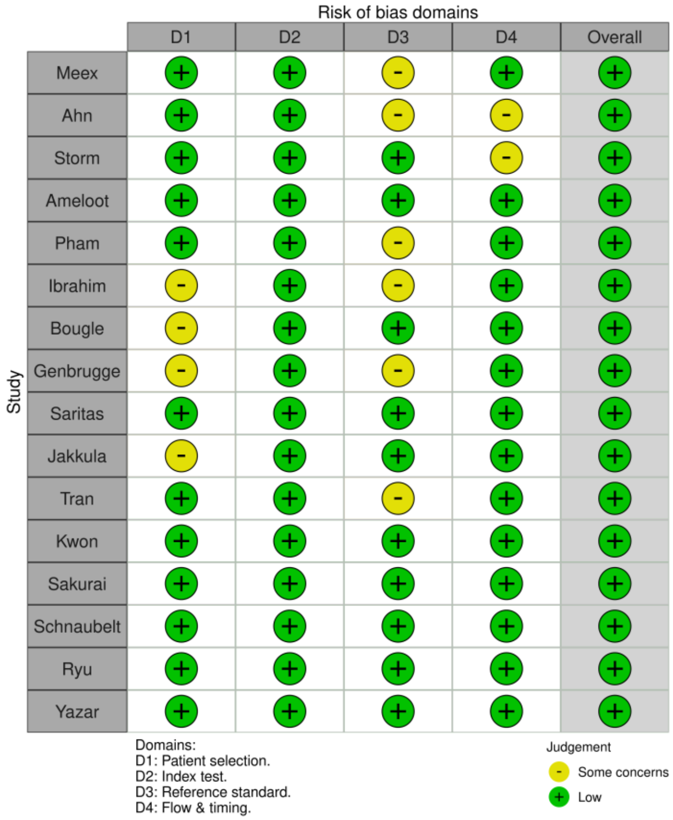

Table e3. Additional characteristics of studies assessing the relationship between cerebral regional oxygen saturation and outcome

Author (Year)	Country	Duration	Product

		Monitoring (Hours)	
Meex[32] (2013)	Belgium	36	Foresight
Ahn[33] (2014)	USA	48	Equanox
Storm[34] (2014)	Germany	40	INVOS
Ameloot[35](2015)	Belgium	24	Foresight
Pham[36] (2015)	Australia	72	Foresight
Ibrahim[37] (2015)	USA	48	INVOS
Bougle[38] (2016)	France	48	INVOS
Genbrugge[39] (2016)	Belgium	48	Foresight
Saritas[40] (2018)	Turkey	5 days	INVOS
Jakkula[41] (2019)	Finland	36	INVOS
Tran[42] (2020)	USA, UK	48	Equanox
Kwon[43](2021)	South Korea	72	INVOS
Sakurai[44] (2020)	Japan	48	INVOS
Schnaubelt[45] (2025)	Austria	72	SenSmart
Ryu[46] (2025)	South Korea	72	INVOS
Yazar[47] (2025)	Turkey	48	INVOS

Figure e4. Forrest plot showing difference in RSO_2 at the start of monitoring between post-cardiac arrest patients with unfavourable versus favourable outcomes.

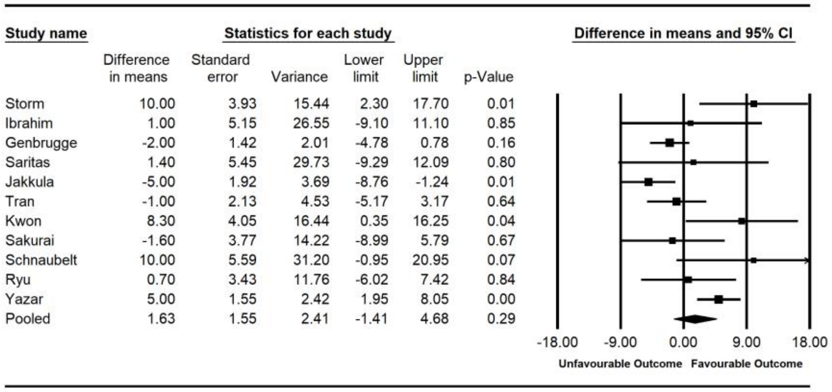

Figure e5. Forrest plot showing difference in mean RSO_2 averaged over the initial 24 hours post-cardiac arrest in patients with unfavourable versus favourable outcomes.

Study name	Difference in means	Standard error	Variance	Lower limit	Upper limit	p-Value
Ahn	5.30	3.05	9.30	-0.68	11.28	0.08
Storm	7.00	2.56	6.54	1.99	12.01	0.01
Pham	-4.00	4.86	23.66	-13.53	5.53	0.41
Bougle	1.10	2.23	4.98	-3.27	5.47	0.62
Genbrugge	1.00	0.69	0.48	-0.35	2.35	0.15
Pooled	2.37	1.41	1.99	-0.40	5.13	0.09

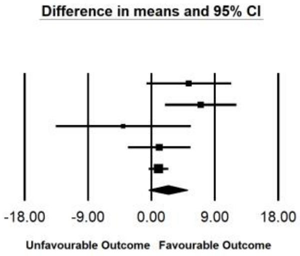

Figure e6. Forrest plot showing difference in mean RSO_2 averaged over 24-48 hours post-cardiac arrest in patients with unfavourable versus favourable outcomes.

Study name	Difference in means	Standard error	Variance	Lower limit	Upper limit	p-Value
Ahn	7.20	3.55	12.59	0.25	14.15	0.04
Storm	9.00	2.62	6.86	3.87	14.13	0.00
Pham	2.00	3.44	11.82	-4.74	8.74	0.56
Bougle	2.30	2.68	7.21	-2.96	7.56	0.39
Pooled	5.23	1.84	3.39	1.62	8.84	0.00

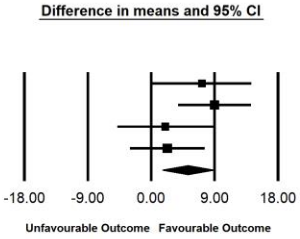

Table e4. Assessment of effect modification for association between rSO_2 and outcomes at 24- and 48-hours post-arrest

Comparison	24 Hours Post-Arrest			48 Hours Post-Arrest		
	Number Studies	Difference in mean rSO_2	P-value for comparison	Number Studies	Difference in mean rSO_2	P-value for comparison
Arrest Duration						
Longer	6	4.5 (0.8-8.1)	0.40	5	2.5 (-0.2-5.2)	0.95
Shorter	5	2.1 (-2.2-6.3)		4	2.3 (-1.0-5.7)	
Proportion Shockable						
Higher	5	3.1 (-1.0-7.2)	0.96	4	2.4 (-0.6-5.3)	0.96
Lower	5	3.9 (-0.4-8.2)		5	2.5 (-0.6-5.6)	
Temperature Goal Initial 24 Hours						
≤ 34°C	6	4.3 (0.7-7.9)	0.46	5	2.6 (-0.2-5.4)	0.87
> 34°C	5	2.3 (-1.8-6.4)		4	2.2 (-1.1-5.5)	
Study Design						
Randomized	2	3.3 (-2.6-9.2)	0.99	1	10.7 (0.9-20.5)	0.08
Observational	9	3.3 (0.6-6.1)		8	1.7 (0.1-3.2)	

Figure e7. Comparison of RSO_2 over time in studies where patients were routinely treated with therapeutic hypothermia (33°C) versus higher temperature targets.

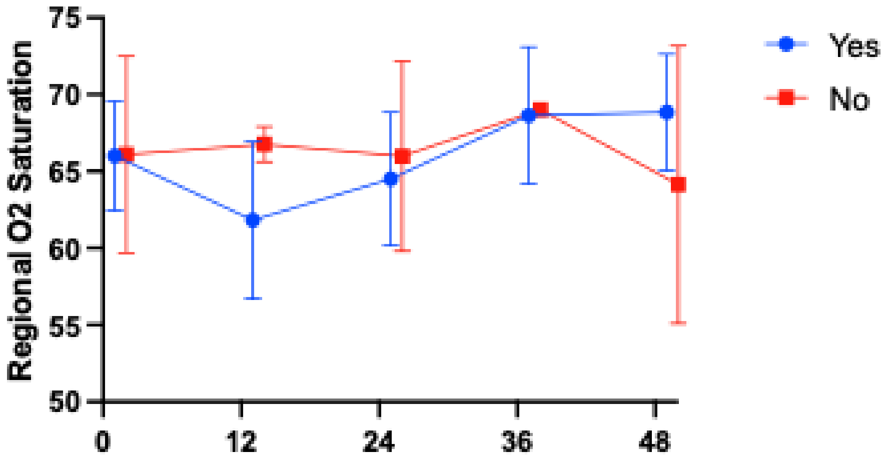

Figure e8. Comparison of RSO_2 over time in studies with a higher versus lower proportions of patients with shockable rhythms.

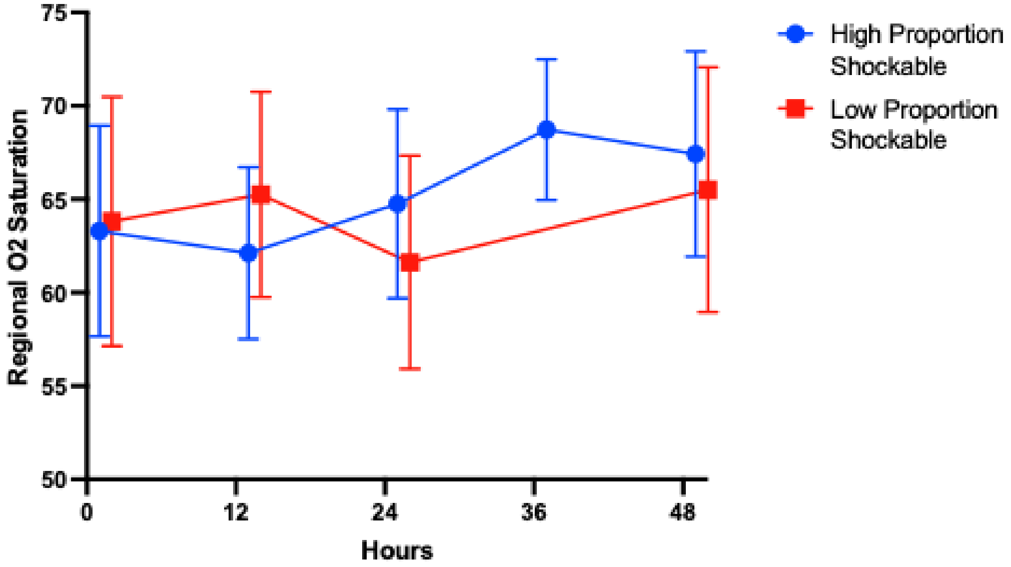

Appendix: Search Results (initially completed May 17, 2024 and updated March 17, 2026)

OVID Medline

#	Searches	Results
1	Heart Arrest/	33138
2	(heart arrest* or cardi* arrest* or 8nglish8* or circulat* arrest* or heart standstill).kf,tw.	62807
3	“Return of Spontaneous Circulation”/	192
4	(return of spontaneous circulation or spontaneous circulation return or ROSC).kf,tw.	5782
5	brain injuries, diffuse/ or brain injury, chronic/ or post-cardiac arrest syndrome/	915
6	brain injur*.kf,tw.	91807
7	post resuscitation.kf,tw.	1490
8	or/1-7	164540
9	Oximetry/	14685
10	oximet*.kf,tw.	17236
11	Oxygen Saturation/	805
12	oxygen saturation.kf,tw.	34841
13	Spectroscopy, Near-Infrared/	16500
14	(near infrared spectroscop* or nir spectroscop* or near infrared spectrometr* or NIRS).kf,tw.	21632
15	hypoxia/ or hypoxia, brain/ or hypoxia-ischemia, brain/	89859
16	(hypoxi* or anoxi* or oxygen deficienc*).kf,tw.	197676
17	or/9-16	288946

18	mortality/ or fatal outcome/ or hospital mortality/ or survival rate/	351400
19	Survival/	4942
20	(mortality or death* or fatality or surviv*).kf,tw.	3032337
21	critical care outcomes/	189
22	critical care outcome*.kf,tw.	374
23	Glasgow Outcome Scale/	2314
24	(Glasgow Outcome Scale or GOS or cerebral performance category or CPC or modified Rankin scale or MRS).kf,tw.	51944
25	or/18-24	3175902
26	8 and 17 and 25	5138
27	limit 26 to 9nglish language	4874
28	exp animals/ not humans/	5221193
29	27 not 28	3704
30	remove duplicates from 29	3692

112 references were added after updating the literature search March 17, 2026

Wiley Cochrane Library

ID	Search	Hits
#1	[mh ^"Heart Arrest"]	1501
#2	(heart arrest* or cardi* arrest* or asystol* or circulat* arrest* or heart standstill):ti,ab,kw	7529
#3	[mh "Return of Spontaneous Circulation"]	14
#4	(return of spontaneous circulation or spontaneous circulation return or ROSC):ti,ab,kw	926
#5	[mh ^"brain injuries, diffuse"] or [mh ^"brain injury, chronic"] or [mh ^"post-cardiac arrest syndrome"]	65
#6	brain injur*:ti,ab,kw	12128
#7	post resuscitation:ti,ab,kw	1619
#8	#1 or #2 or #3 or #4 or #5 or #6 or #7	20364
#9	[mh ^Oximetry]	1122
#10	oximet*:ti,ab,kw	7594
#11	[mh "Oxygen Saturation"]	98
#12	oxygen saturation:ti,ab,kw	18528
#13	[mh "Spectroscopy, Near-Infrared"]	539
#14	("near infrared" NEXT spectroscop*):ti,ab,kw or (nir NEXT spectroscop*):ti,ab,kw or ("near infrared" NEXT spectrometr*):ti,ab,kw or NIRS:ti,ab,kw	2771

#15	[mh ^hypoxia] or [mh ^"hypoxia, brain"] or [mh ^"hypoxia-ischemia, brain"]	3348
#16	(hypoxi* or anoxi* or oxygen deficienc*):ti,ab,kw	11039
#17	#9 or #10 or #11 or #12 or #13 or #14 or #15 or #16	33612
#18	[mh ^mortality] or [mh ^"fatal outcome"] or [mh ^"hospital mortality"] or [mh ^"survival rate"]	16314
#19	[mh Survival]	183
#20	(mortality or death* or fatality or surviv*):ti,ab,kw	275844
#21	[mh "critical care outcomes"]	11
#22	critical care outcome*:ti,ab,kw	17223
#23	[mh "Glasgow Outcome Scale"]	192
#24	(Glasgow Outcome Scale or GOS or cerebral performance category or CPC or modified Rankin scale or MRS):ti,ab,kw	11549
#25	#18 or #19 or #20 or #21 or #22 or #23 or #24	292400
#26	#8 and #17 and #25	829
#27	Cochrane Reviews	31
	Trials	796

WHO International Clinical Trials Registry Platform (ICTRP) Search Portal

For all searches: Recruitment Status is ALL; With Results Only.

Search 1

heart arrest* or cardi* arrest* or asystol* or circulat* arrest* or heart standstill or return of spontaneous circulation or spontaneous circulation return or ROSC or brain injur* or post resuscitation	in the Title

AND

oximet*	in the Intervention

Search 2

heart arrest* or cardi* arrest* or asystol* or circulat* arrest* or heart standstill or return of spontaneous circulation or spontaneous circulation return or ROSC or brain injur* or post resuscitation	in the Title

AND

oxygen saturation	in the Condition

Search 3

heart arrest* or cardi* arrest* or asystol* or circulat* arrest* or heart standstill or return of spontaneous circulation or spontaneous circulation return or ROSC or brain injur* or post resuscitation	in the Title

AND

near infrared spectroscop*	in the Intervention

Search 3

heart arrest* or cardi* arrest* or asystol* or circulat* arrest* or heart standstill or return of spontaneous circulation or spontaneous circulation return or ROSC or brain injur* or post resuscitation	in the Title

AND

nir spectroscop*	in the Intervention

Search 4

heart arrest* or cardi* arrest* or asystol* or circulat* arrest* or heart standstill or return of spontaneous circulation or spontaneous circulation return or ROSC or brain injur* or post resuscitation	in the Title

AND

near infrared spectrometr*	in the Intervention

Search 5

heart arrest* or cardi* arrest* or asystol* or circulat* arrest* or heart standstill or return of spontaneous circulation or spontaneous circulation return or ROSC or brain injur* or post resuscitation	in the Title

AND

NIRS	in the Intervention

Search 6

heart arrest* or cardi* arrest* or asystol* or circulat* arrest* or heart standstill or return of spontaneous circulation or spontaneous circulation return or ROSC or brain injur* or post resuscitation	in the Title

AND

hypoxi*	in the Condition

Search 7

heart arrest* or cardi* arrest* or asystol* or circulat* arrest* or heart standstill or return of spontaneous circulation or spontaneous circulation return or ROSC or brain injur* or post resuscitation	in the Title

AND

anoxi*	in the Condition

Search 8

heart arrest* or cardi* arrest* or asystol* or circulat* arrest* or heart standstill or return of spontaneous circulation or spontaneous circulation return or ROSC or brain injur* or post resuscitation	in the Title

AND

oxygen deficienc*	in the Condition

Database(s): **Embase** 1974 to 2024 May 16
Search Strategy:
https://ezproxy.lib.ucalgary.ca/login?url=https://ovidsp.ovid.com/ovidweb.cgi?T=JS&NEWS=N&PAGE=main&SHAREDSEARCHID=1kpPfsPVXEnagZtMyvdnMQpe9jnxszdiVU0UUJcvAorlAn1jHiToWxhyoRt8zYlpk

#	Searches	Results
1	heart arrest/ or cardiopulmonary arrest/ or "out of hospital cardiac arrest"/	109457
2	(heart arrest* or cardi* arrest* or asystol* or circulat* arrest* or heart standstill).kf,tw.	96977
3	"return of spontaneous circulation"/	12568
4	(return of spontaneous circulation or spontaneous circulation return or ROSC).kf,tw.	11576
5	brain damage/ or diffuse brain injury/ or post-cardiac arrest syndrome/	43890
6	brain injur*.kf,tw.	129446
7	post resuscitation.kf,tw.	2693
8	or/1-7	300729
9	oximetry/	11843
10	oximet*.kf,tw.	24832
11	oxygen saturation/	87602
12	oxygen saturation.kf,tw.	53017
13	near infrared spectroscopy/	27796
14	(near infrared spectroscop* or nir spectroscop* or near infrared spectrometr* or NIRS).kf,tw.	26531

15	hypoxia/ or brain hypoxia/	149843
16	(hypoxi* or anoxi* or oxygen deficienc*).kf,tw.	267179
17	or/9-16	442901
18	mortality/ or all cause mortality/ or cardiovascular mortality/ or hospital mortality/ or mortality rate/	1194043
19	survival/	334757
20	(mortality or death* or fatality or surviv*).kf,tw.	4348905
21	critical care outcome/	573
22	critical care outcome*.kf,tw.	567
23	Glasgow outcome scale/	7970
24	(Glasgow Outcome Scale or GOS or cerebral performance category or CPC or modified Rankin scale or MRS).kf,tw.	85213
25	or/18-24	4670945
26	8 and 17 and 25	10592
27	limit 26 to english language	10195
28	exp animal/ not exp human/	5254562
29	27 not 28	8351

Database: Embase <1974 to 2026 March 17>

Search Strategy:
1 heart arrest/ or cardiopulmonary arrest/ or "out of hospital cardiac arrest"/ (129625)
2 (heart arrest* or cardi* arrest* or asystol* or circulat* arrest* or heart standstill).kf,tw. (112047)
3 "return of spontaneous circulation"/ (15026)
4 (return of spontaneous circulation or spontaneous circulation return or ROSC).kf,tw. (13860)
5 brain damage/ or diffuse brain injury/ or post-cardiac arrest syndrome/ (51613)
6 brain injur*.kf,tw. (150673)
7 post resuscitation.kf,tw. (3189)
8 or/1-7 (347582)
9 oximetry/ (13706)
10 oximet*.kf,tw. (30920)
11 oxygen saturation/ (119937)
12 oxygen saturation.kf,tw. (65128)
13 near infrared spectroscopy/ (33629)
14 (near infrared spectroscop* or nir spectroscop* or near infrared spectrometr* or NIRS).kf,tw. (32616)
15 hypoxia/ or brain hypoxia/ (176462)
16 (hypoxi* or anoxi* or oxygen deficienc*).kf,tw. (302253)
17 or/9-16 (522350)
18 mortality/ or all cause mortality/ or cardiovascular mortality/ or hospital mortality/ or mortality rate/ (1390262)
19 survival/ (344772)
20 (mortality or death* or fatality or surviv*).kf,tw. (5023530)
21 critical care outcome/ (783)
22 critical care outcome*.kf,tw. (736)
23 Glasgow outcome scale/ (9639)
24 (Glasgow Outcome Scale or GOS or cerebral performance category or CPC or modified Rankin scale or MRS).kf,tw. (101582)
25 or/18-24 (5382896)
26 8 and 17 and 25 (13098)
27 limit 26 to english language (12687)
28 exp animal/ not exp human/ (5943564)
29 27 not 28 (10553)
30 limit 29 to (human and english language and yr="2024 - 2026") (1892)
31 limit 30 to yr="2024 - 2026" (1892)
32 limit 31 to (human and english language and (clinical trial or randomized controlled trial or controlled clinical trial or multicenter study or phase 1 clinical trial or phase 2 clinical trial or phase 3 clinical trial or phase 4 clinical trial) and yr="2024 - 2026" and (adult or aged) and last 2 years) (198)
33 from 31 keep 1-1892 (1892)
